# Supplementary material for: Tspan8 Drives Melanoma Dermal Invasion by Promoting ProMMP-9 Activation and Basement Membrane Proteolysis in a Keratinocyte-Dependent Manner
Source: Cancers (Basel). 2020 May 21;12(5):1297. doi: 10.3390/cancers12051297 (PMC7281247; doi:10.3390/cancers12051297)
Supplement: Supplementary file 1 [file cancers-12-01297-s001.pdf]

Supplementary Materials: Tspan8 Drives Melanoma Dermal Invasion by Promoting ProMMP-9 Activation and Basement Membrane Proteolysis in a Keratinocyte-Dependent Manner

Manale El Kharbili, Muriel Cario, Nicolas Béchetoille, Catherine Pain, Claude Boucheix, Françoise Degoul, Ingrid Masse and Odile Berthier-Vergnes

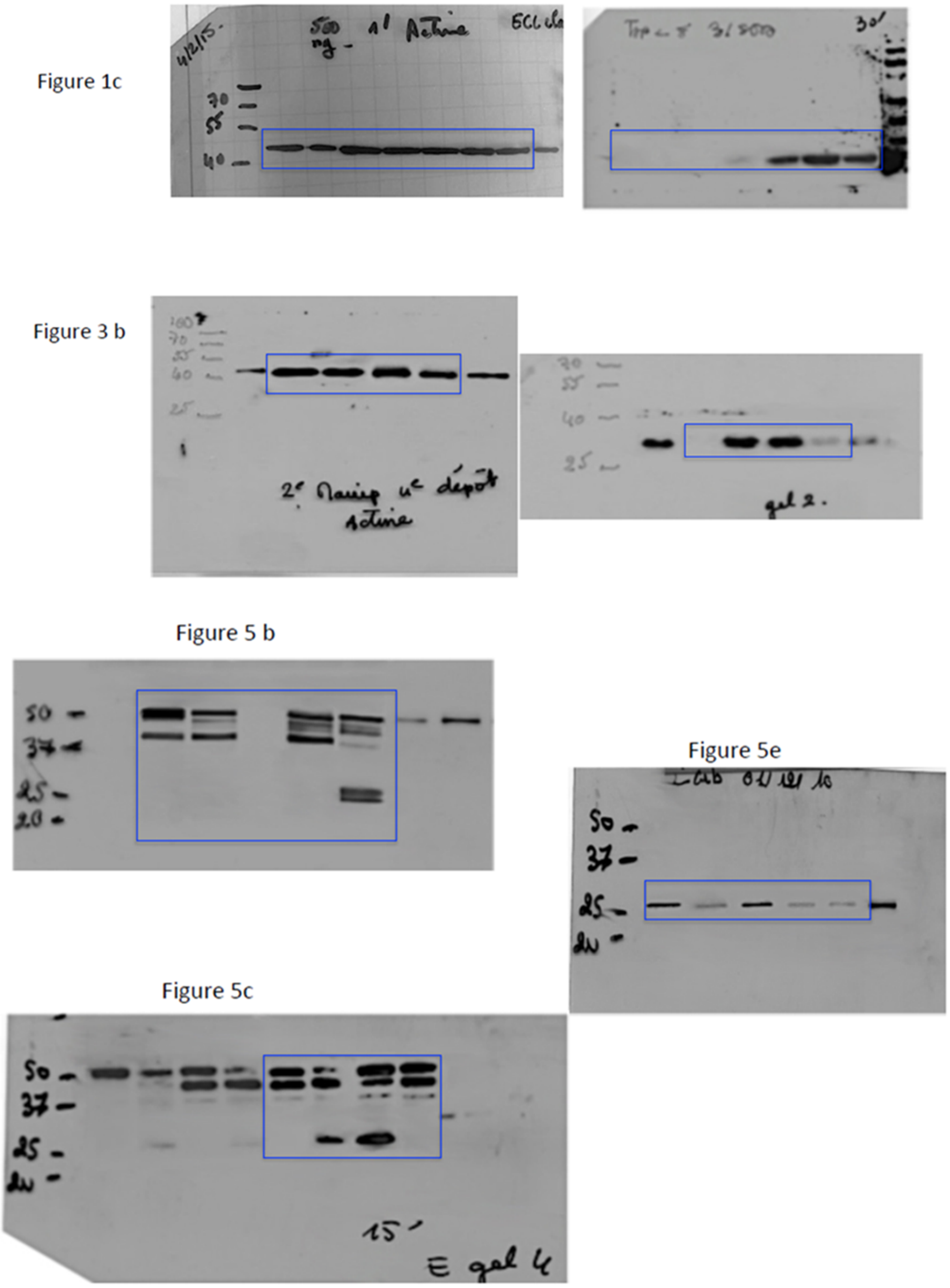

Figure S1. Uncropped western blot figures.

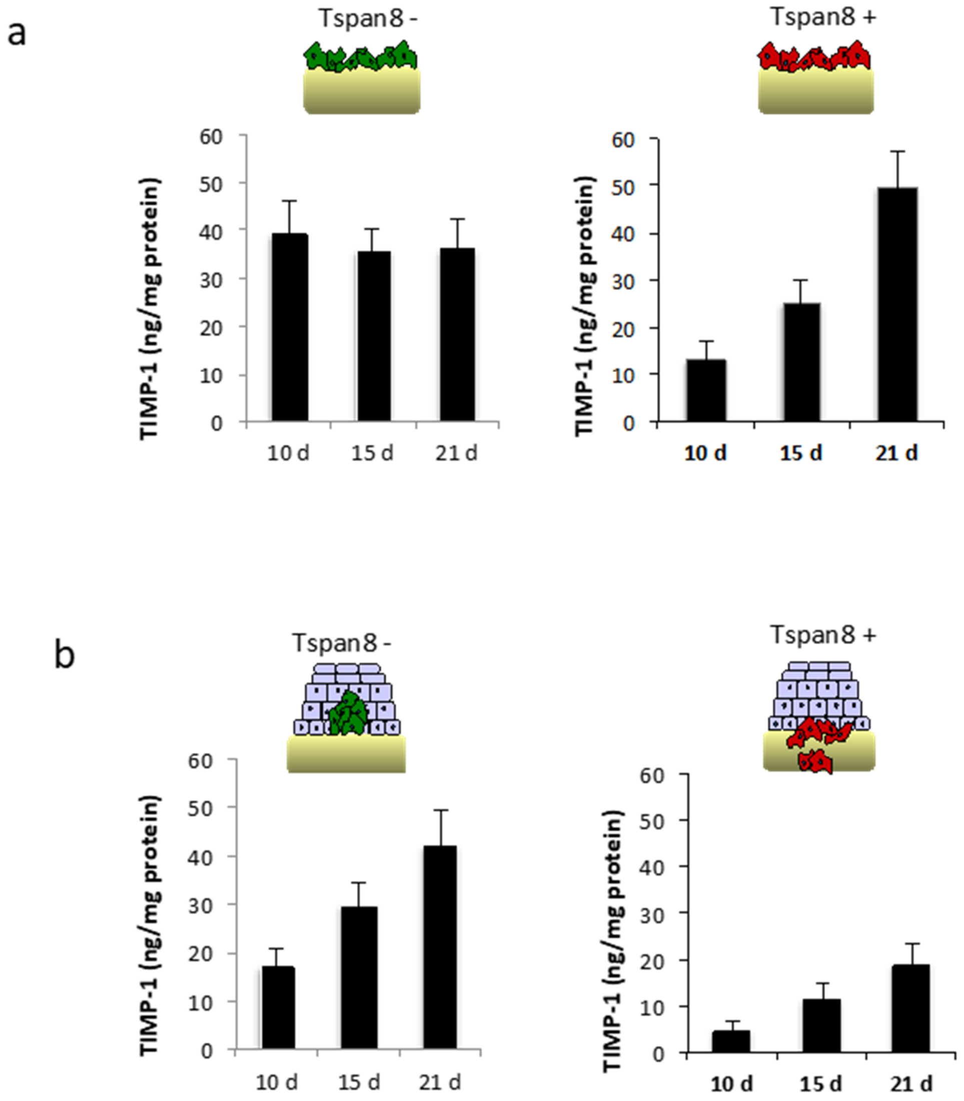

Figure S2. Low TIMP-1 levels were exclusively observed when melanoma cells expressed Tspan8 and were integrated into SR. (**a**,**b**) Supernatants TIMP-1 protein levels were measured at day 10, 15 and 21 by ELISA in composites consisting of melanoma cells expressing or not Tspan8 cultured on acellular dermis either alone (**a**) or with human keratinocytes (**b**). Results are represented as the mean ± SEM from three independent experiments.

© 2020 by the authors. Submitted for possible open access publication under the terms and conditions of the Creative Commons Attribution (CC BY) license (http://creativecommons.org/licenses/by/4.0/).
